# Supplementary material for: Cyclophilin A‐mediated cis/trans isomerization modulates RIN4 to control intracellular rhizobial infection in legumes
Source: New Phytol. 2026 Apr 4;250(6):3932–45. doi: 10.1111/nph.71147 (PMC13193524; doi:10.1111/nph.71147)
Supplement: Supplementary file 1 — Fig. S1 Sequential and structural alignment of LjCyPAs and AtROC1. Fig. S2 mRNA abundance of LjCyPA1, LjCyPA2, and LjCyPA3 in early‐infection stage. Fig. S3 Schematic illustration of CRISPR/Cas9‐induced nonsense mutation in LjCyPAs. Fig. S4 Number of infection threads in MG‐20 wild‐type and cypA1 another mutant allele (LjcypA1‐2). Fig. S5 Infection thread numbers in LjcypA1‐1 mutant are restored in hairy roots expressing LjCyPA1. Fig. S6 High‐resolution images of abnormal infection threads at the elongation stages in LjcypA1‐1 mutant. Fig. S7 Transcriptomic differences in response to inoculation with M. loti ΔT3SS mutant in MG‐20 and LjcypA1‐1 mutant. Fig. S8 Quantitative RT‐PCR analysis of immune‐/defense‐related and symbiotic gene expression in MG‐20 and LjcypA1‐1 mutant, with or without M. loti MAFF303099 or its ΔT3SS mutant. Fig. S9 Gain‐of‐function of LjCyPA1 promotes symbiosis with M. loti MAFF303099 and the M. loti ΔT3SS. Fig. S10 Bimolecular fluorescence complementation between LjCyPA1 and LjRIN4. Fig. S11 CyPA2 and CyPA3 conservation in representative legumes and nonlegumes. Table S1 Gene IDs and names of the T3SS cluster deleted in this study for mutant construction. Table S2 List of primers. [file NPH-250-3932-s002.pdf]

New Phytologist Supporting Information

Cyclophilin A-mediated cis/trans isomerization modulates RIN4 to control intracellular rhizobial infection in legumes

Takashi Goto, Kasper Røjkjær Andersen, Masaru Bamba, Shusei Sato, Masayuki Sugawara, Kiwamu Minamisawa, Masayoshi Kawaguchi, Jens Stougaard, and Yasuyuki Kawaharada

Article acceptance date: 13 March 2026

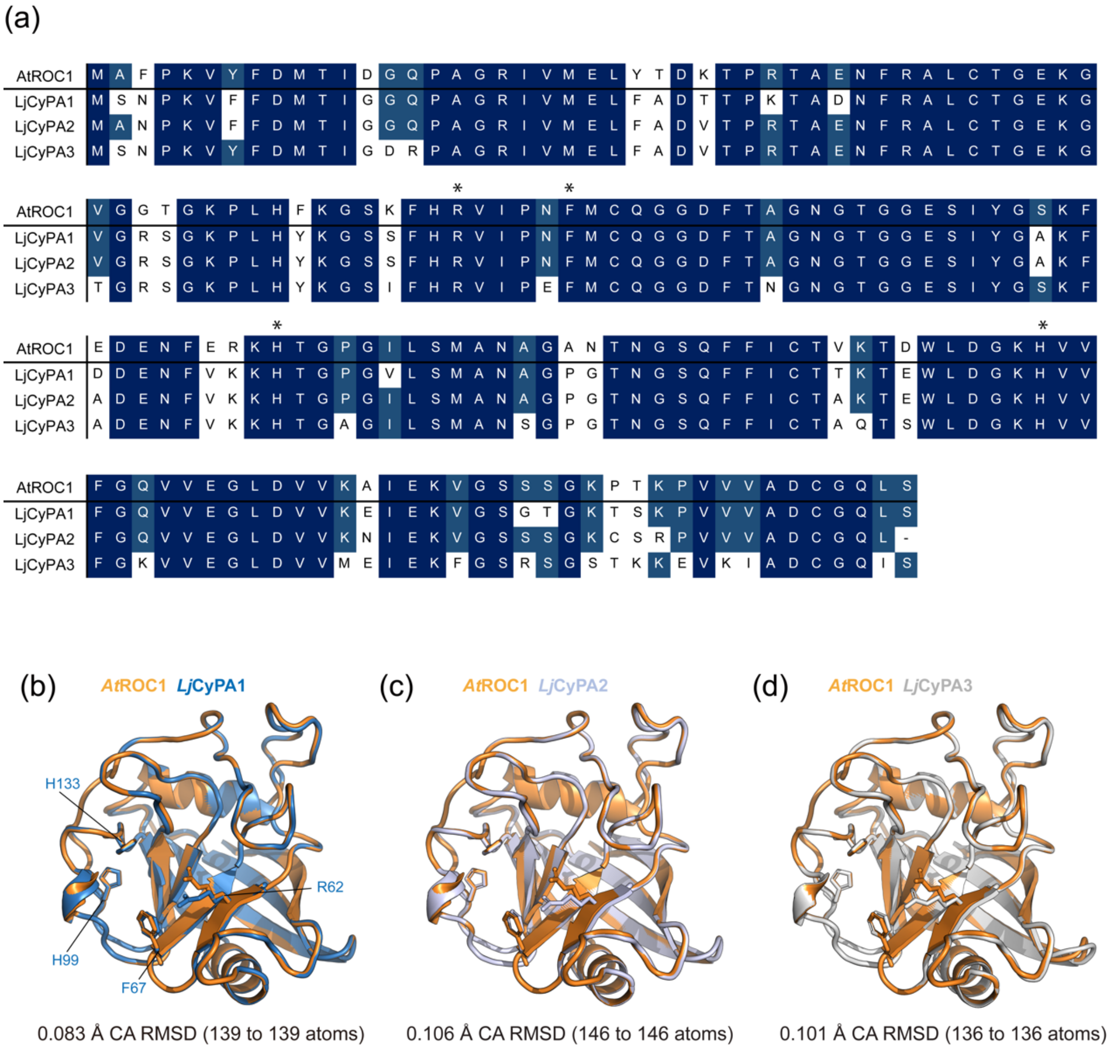

Supplementary figure 1. Sequential and structural alignment of LjCyPAs and AtROC1. (a) Amino acid sequences of full-length AtROC1 (At4g38740), LjCyPA1 (Lj1g3v3343880), LjCyPA2 (Lj3g3v3527420), and LjCyPA3 (Lj3g3v3527430). Important residues for the catalytic function are highlighted in asterisks. (b-d) Structural alignment of each LjCyPA with AtROC1. Superposition of each LjCyPA (blue) and AtROC1 (orange) structural models with a root-mean-square deviation (RMSD). Important residues for the catalytic function are highlighted in b.

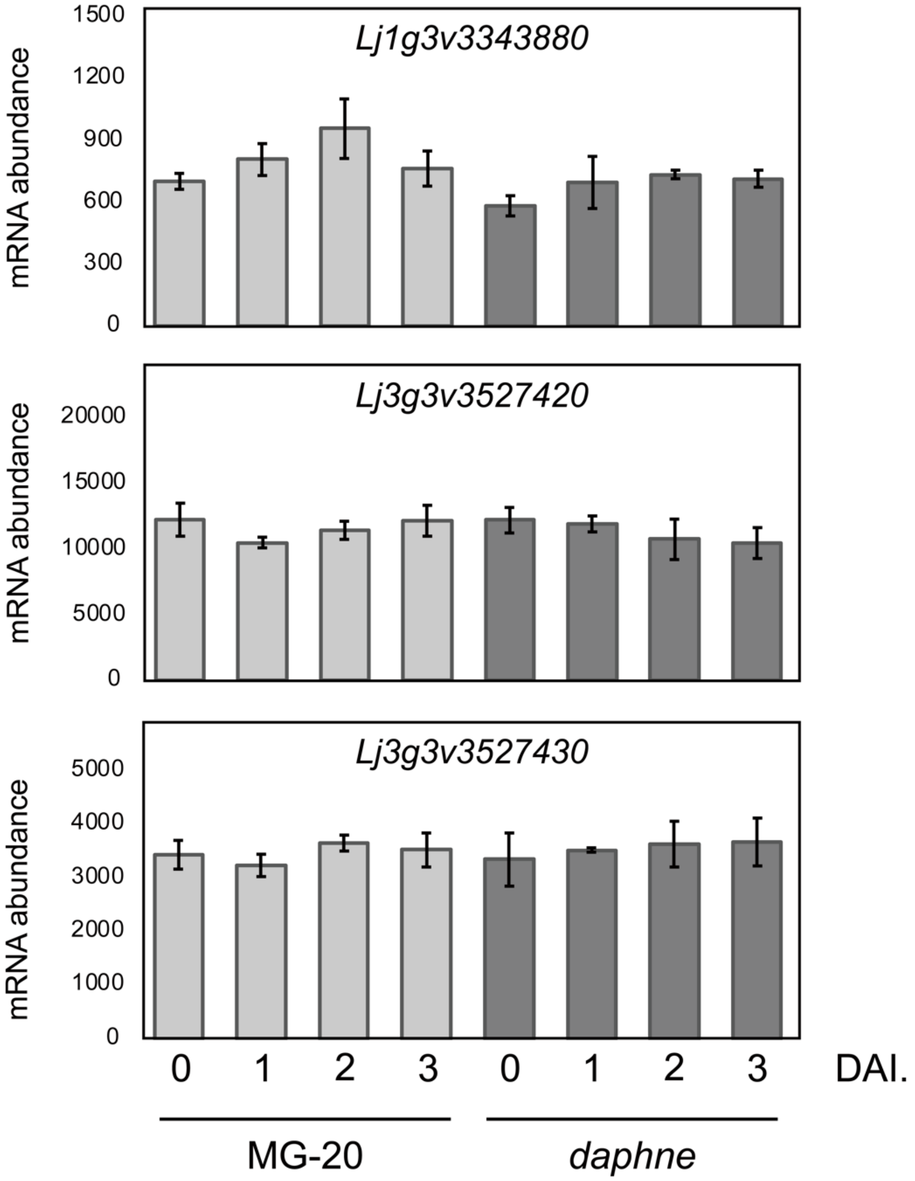

Supplementary figure 2. mRNA abundance of *LjCyPA1*, *LjCyPA2,* and *LjCyPA3* in early infection stage. MG-20 (wild-type; light-gray) and *daphne* (mutant which shows excessive infection of rhizobia; dark-gray) at 0 (non-inoculation), 1, 2, and 3 DAI. Error bars indicate means ± SDs. (*n = 20 roots* for each biological replicate).

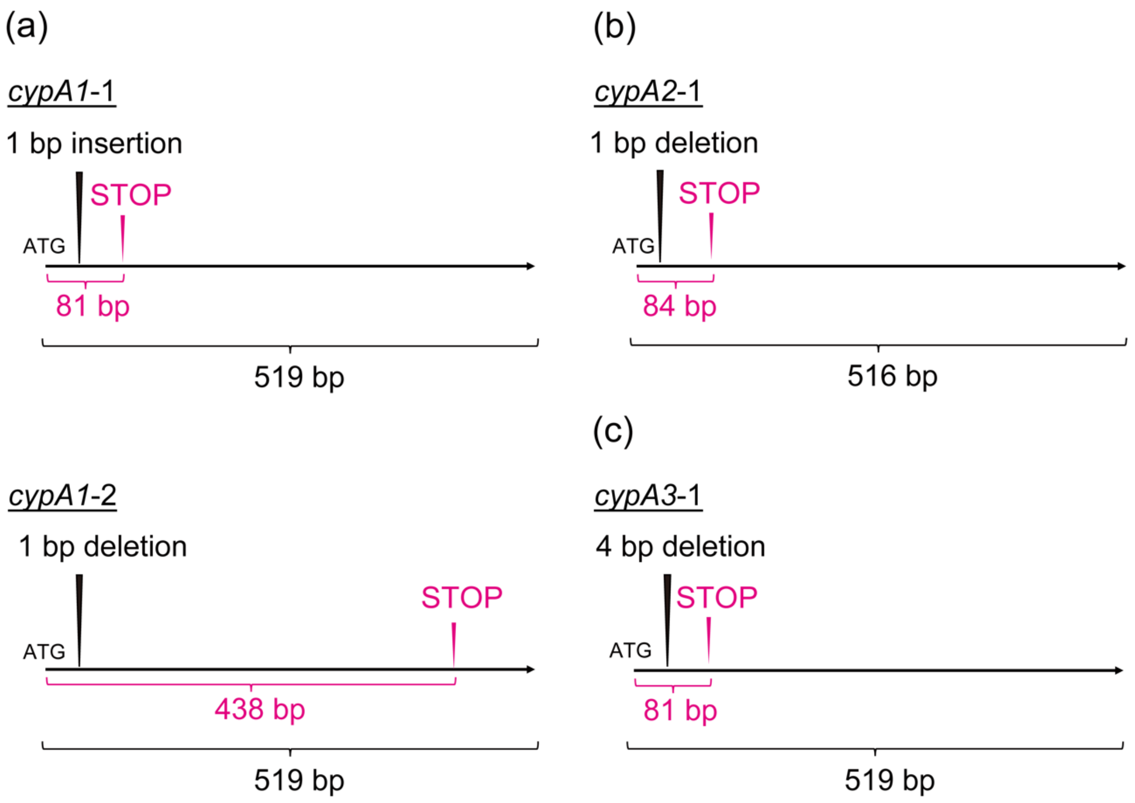

Supplementary figure 3. Schematic illustration of CRISPR/Cas9-induced nonsense mutation in *LjCyPAs*. Each gRNA induces the insertion or deletion of several base pairs, causing a frameshift. The black arrowhead indicates the site where the insertion or deletion occurs, and the pink color represents the newly formed stop codon.

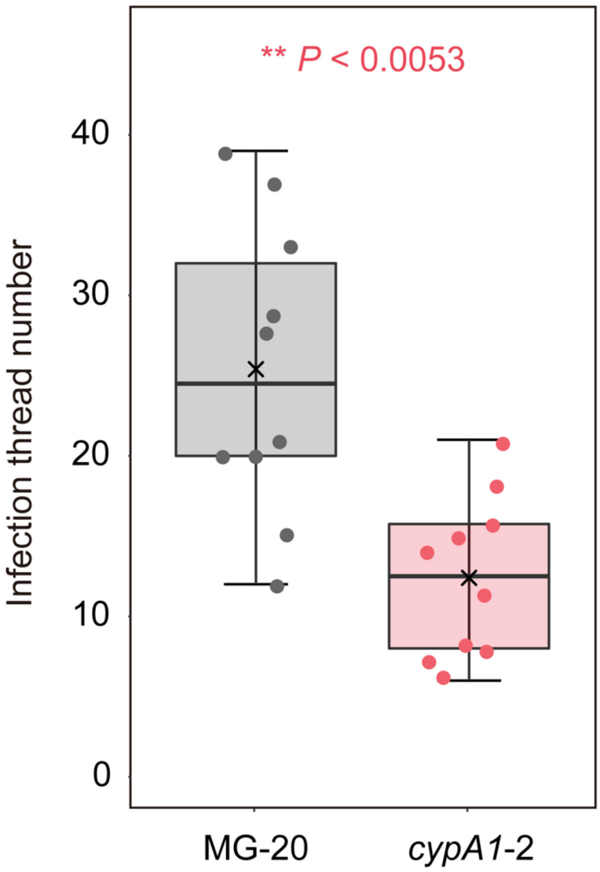

Supplementary figure 4. The number of infection threads in MG-20 wild-type and *cypA1* another mutant allele (*LjcypA1-2*). Each dot represents the number of infection threads of each plant. *n = 10* (MG-20 and *LjcypA1-2*). Asterisks indicate that differences are statistically significant (Welch's t test).

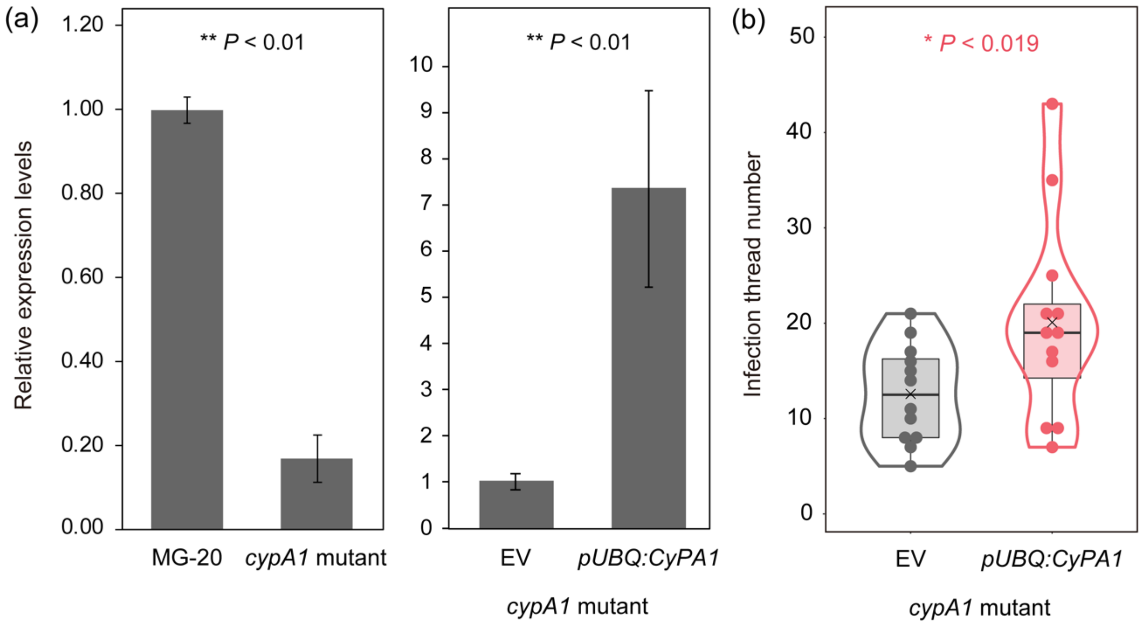

Supplementary figure 5. Infection thread numbers in *LjcypA1-1* mutant are restored in hairy roots expressing *LjCyPA1*. (a) Quantitative RT-PCR analysis of *LjCyPA1* expression in MG-20 and the *cypA1* mutant (left) and EV and *pUBQ:LjCyPA1* in the *cypA1* hairy roots (right). Error bars indicate the mean ± SSD. (n = 10 roots per biological replicate). Asterisks indicate statistical difference (Welch's t-test). (b) Each dot represents the number of infection threads of each *cypA1* roots in control (empty vector; EV) and constitutive expression of *LjCyPA1* (*pUBQ:LjCyPA1*). n = 12 roots for EV and *pUBQ:LjCyPA1*.

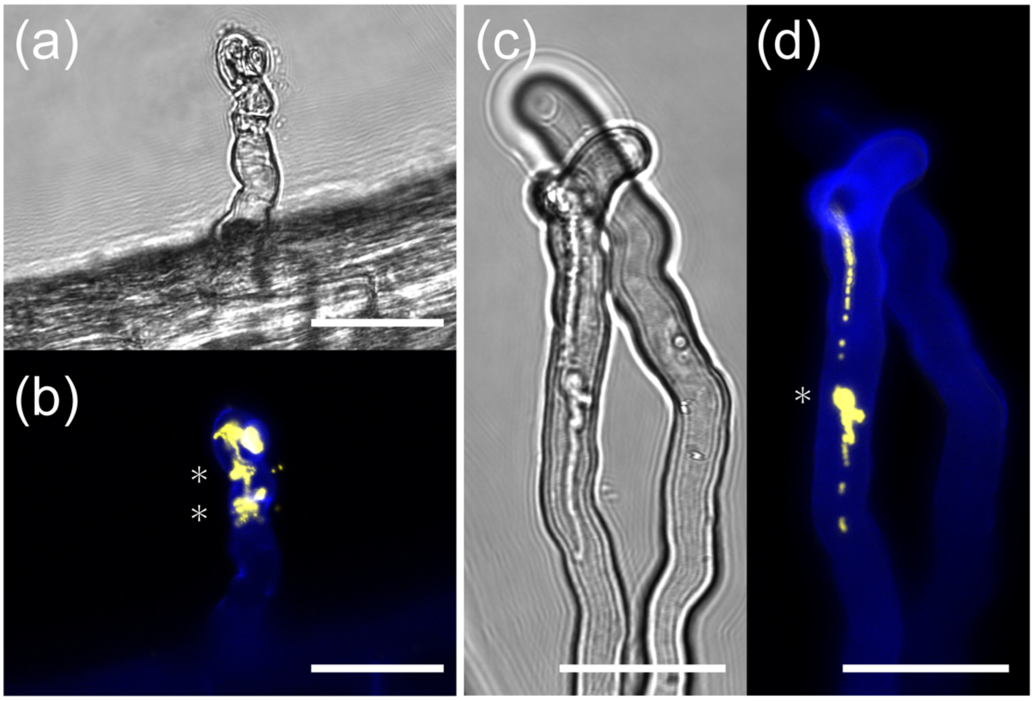

Supplementary figure 6. High-resolution images of abnormal infection threads at the elongation stages in *LjcypA1-1* mutant. (a and c) Bright images show infection thread structure. (c and d) Fluorescence images show *M. loti MAFF303099*. Asterisks indicate enlarged bulbs. Scale bars represent 50 μm.

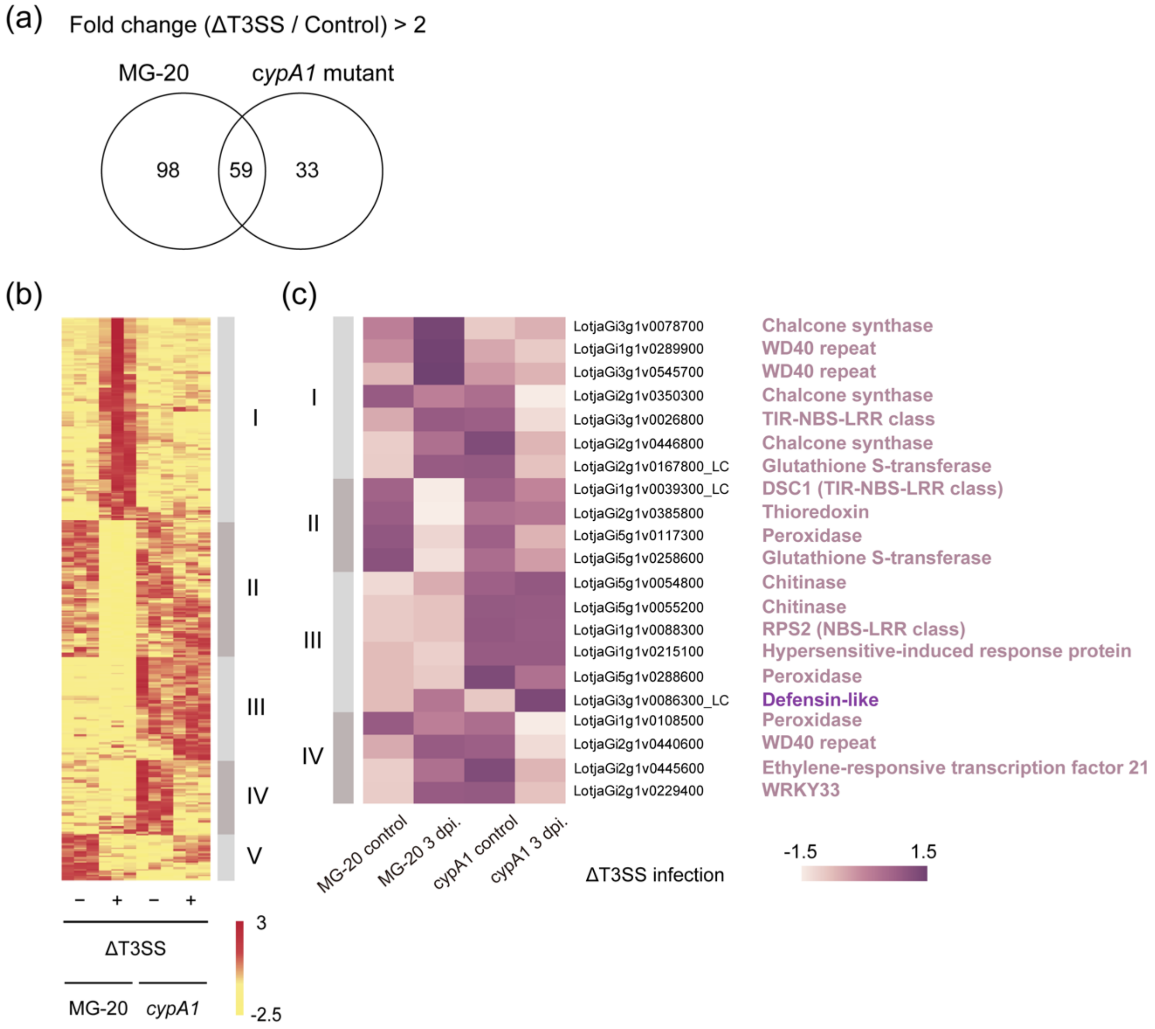

Supplementary figure 7. Transcriptomic differences in response to inoculation with *M. loti* ΔT3SS mutant in MG-20 and *LjcypA1-1* mutant. (a) Venn diagram showing the overlap of differentially expressed genes (fold change > 2 relative to the non-inoculated control) in MG-20 and the *cypA1* mutant after ΔT3SS inoculation. (b) Heat map along with hierarchical clustering of the differentially expressed genes. Colors indicate Z-score–normalized expression levels. (c) Expression patterns of resistance- and defense-related genes in each cluster defined in (b). A defensin-like gene, shown in dark purple, exhibits synergistic effects of the T3SS and *cypA1* mutations.

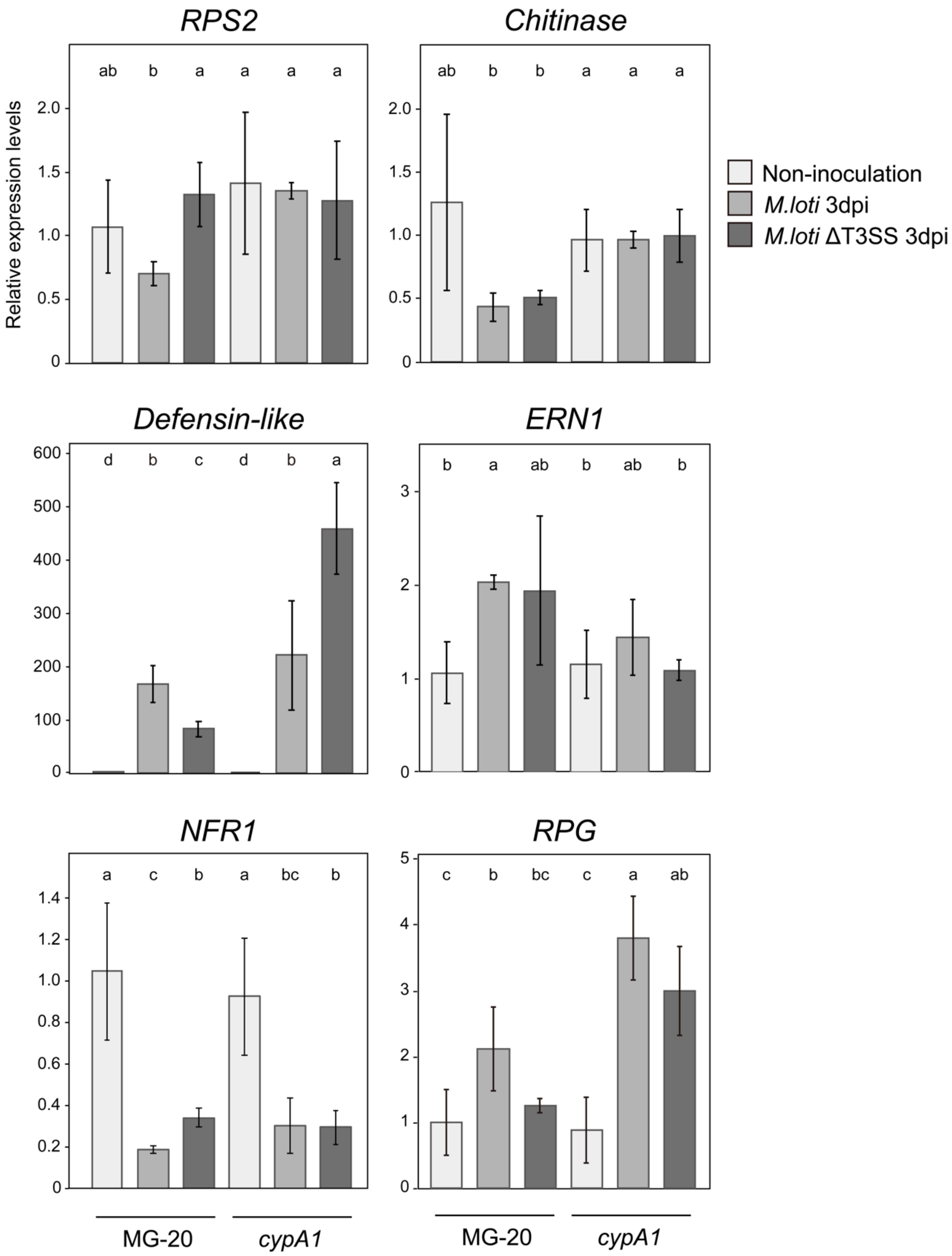

Supplementary figure 8. Quantitative RT-PCR analysis of immune-/defense-related and symbiotic gene expression in MG-20 and *LjcypA1-1* mutant, with or without *M.*

***loti* MAFF303099 or its ΔT3SS mutant.** MG-20 (left) and the *cypA1* mutant (right) under mock conditions (non-inoculated, white) or 3 days after inoculation with *M. loti* MAFF303099 (light gray) or its ΔT3SS mutant (dark gray). Error bars indicate the mean ± SD. (*n* = *10 roots* per biological replicate). ANOVA followed by Tukey's HSD test (P < 0.05). Different letters indicate statistically significant differences.

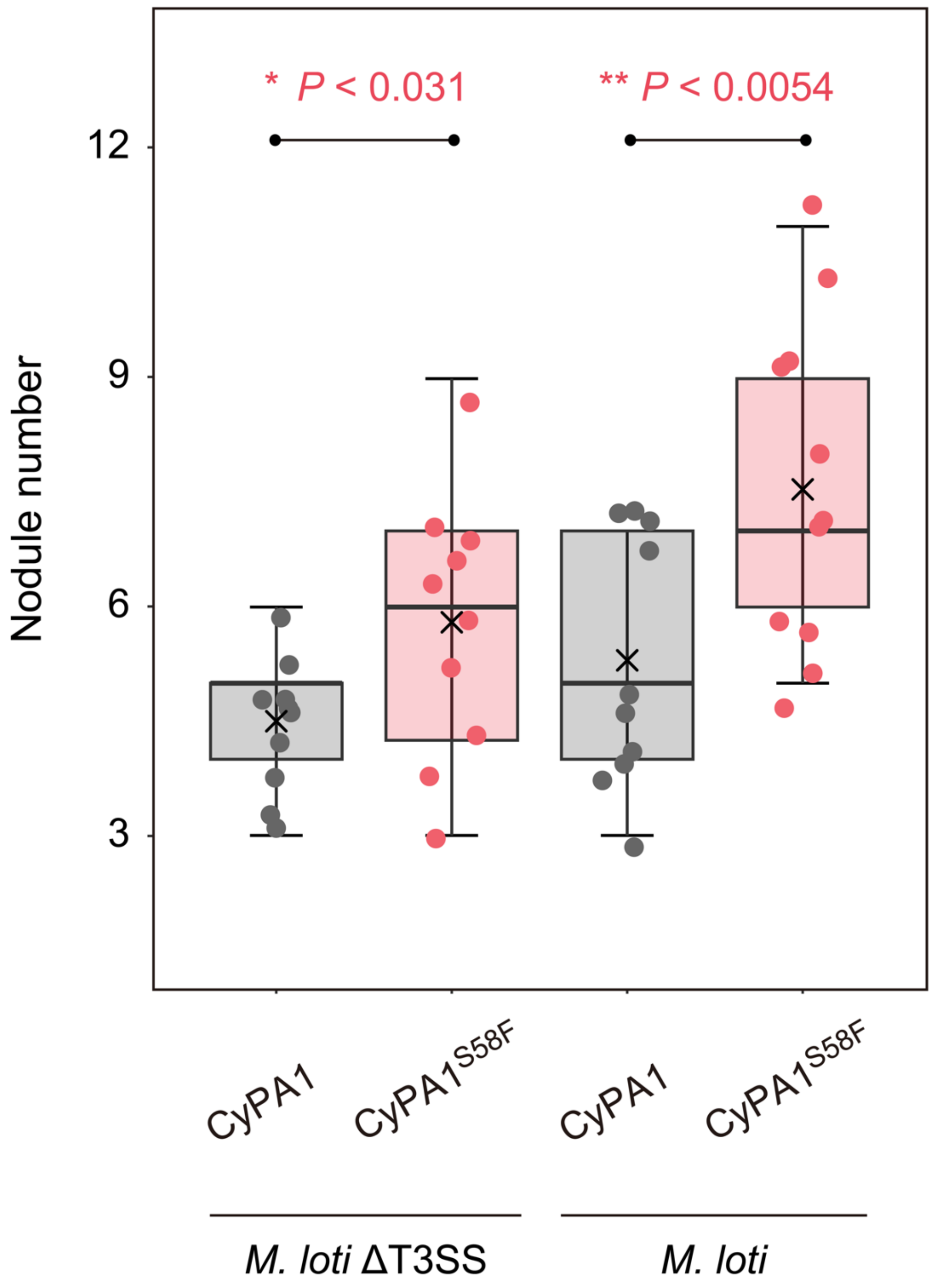

Supplementary figure 9. Gain-of-function of *LjCyPA1* promotes symbiosis with *M. loti* MAFF303099 and the *M. loti* ΔT3SS. The number of nodules in MG-20 hairy roots harboring *pUBQ:LjCyPA1-GFP* vector (control; gray) and *pUBQ:CyPA1*^{S58F}*-GFP* vector (pink) 3 weeks after inoculation. Asterisks indicate statistical difference by Welch's t-test.

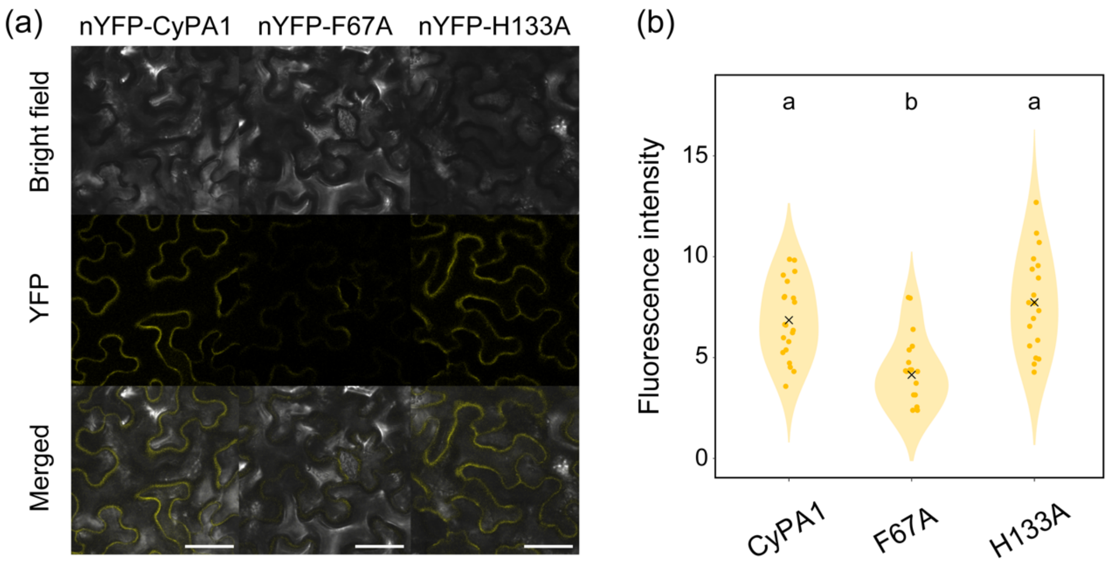

Supplementary figure 10. Bimolecular fluorescence complementation between LjCyPA1 and LjRIN4. (a) Bright field and YFP fluorescence in tobacco leaves co-expressing nYFP-LjCyPA1 or its variants (F67A and H133A) with cYFP-LjRIN4. Scale bars represent 50 µm. (b) Quantification of fluorescence intensity for nYFP-LjCyPA1 and its variants (F67A and H133A) co-expressed with cYFP-LjRIN4. Statistical analysis was performed using ANOVA followed by Tukey's HSD test ($P < 0.05$). Different letters indicate statistically significant differences.

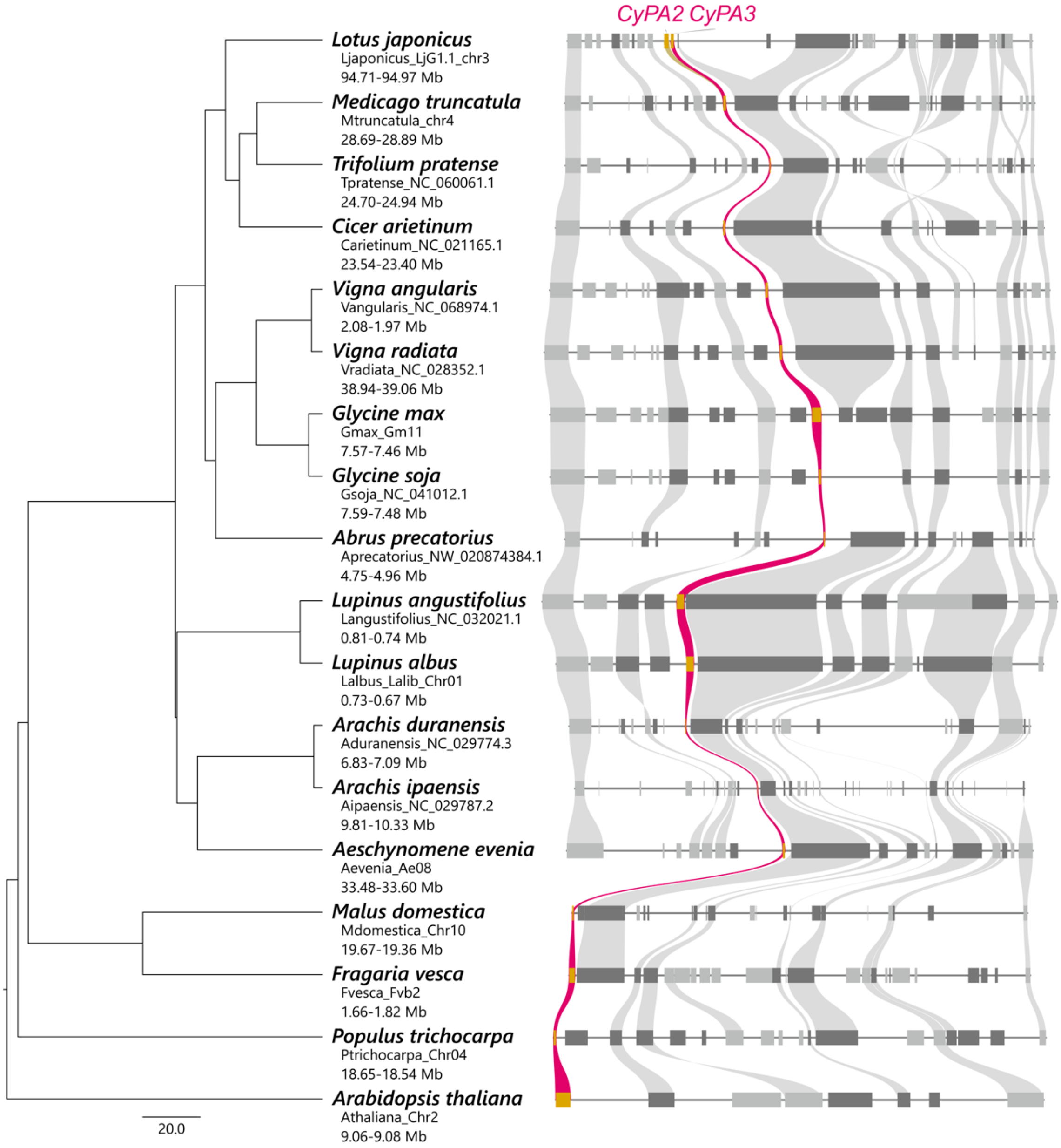

Supplementary figure 11. *CyPA2* and *CyPA3* conservation in representative legumes and non-legumes. Orthologous genes in each specific block are connected by lines of gray or pink colors. *CyPA2/3* orthologues are highlighted in pink. Dark gray represents genes on the minus strand, while light gray represents genes on the plus strand. The phylogenetic tree to the left of the synteny blocks was obtained from TimeTree5, with the scale bar indicating divergence time in million years ago (Mya).

Supplementary table 1. Gene IDs and names of the T3SS cluster deleted in this study for mutant construction

Gene ID	Name
mlr6342	*rhcN*
mlr6343	*y4yj*
mlr6344	*rhcQ*
mlr8766	*rhcR*
smr8694	*rhcS*
mlr6345	*rhcT*
mlr6346	*rhcU*

Supplementary table 2. List of primers

Name	Sequence
Oligonucleotides for CRISPR/Cas9 mutagenesis<br>* The underlined 4-bp sequence for ligation	
gRNA-1F	5'- ATTGGTCTTCTTCGACATGACCAT -3'
gRNA-1R	5'- AAACATGGTCATGTCGAAGAAGAC -3'
gRNA-2F	5'- ATTGTCATGTCGAAGAAAACCTTA -3'
gRNA-2R	5'- AAACTAAGGTTTTCTTCGACATGA -3'
gRNA-3F	5'- ATTGCCATGACGATGCGACCGGCG -3'
gRNA-3R	5'- AAACCGCCGGTCGCATCGTCATGG -3'
Primers for cloning of CDS of CyPA and RIN4<br>* The underlined sequences for pENTR/D-TOPO or BP reaction	
CyPA1-F	5'- CACCATGTCTAACCCTAAGGTCTTCTTCG -3'
CyPA1-R	5'- CTACGAAAGTTGACCGCAATCG -3'
RIN4-F	5'- ACAAGTTTGTACAAAAAAGCAGGCT<br>ATGGCACAACGTTCTCATG -3'
RIN4-R	5'- ACCACTTTGTACAAGAAAGCTGGGT<br>TCATTTCTTGCTAAACCCAAAG -3'

Site-directed mutagenesis by PrimeSTAR® Mutagenesis<br>＊ The underlined sequences for the mutation sites			
CyPA1-S58F-F	5’ –	AAGGGCTTTTCCTTCCACCGTGTCATC	–3’
CyPA1-S58F-R	5’ –	GAAGGAAAAGCCCTTGTAGTGGAGAGG	–3’
RIN4-P149V-F	5’ –	GCTGTTGTGAAGTTTGGTGAGTGGGAC	–3’
RIN4-P149V-R	5’ –	AAACTTCACAACAGCAGCACCTTTCTC	–3’
RIN4-ΔP149-F	5’ –	GCTGTT__AAGTTTGGTGAGTGGGAC	–3’
RIN4-ΔP149-R	5’ –	AAACTT__AACAGCAGCACCTTTCTC	–3’
Primers for BIFC construction by NEBuilder® HiFi DNA Assembly			
VYNE-CyPA1-F1	5’ –	ACAAAAAAGCAGGCTATGGTGAGCAAGGGCGAGG	–3’
VYNE-CyPA1-R1	5’ –	CTTAGGGTTAGACATACCTCCAAGATCCTCCTCAGAAATC	–3’
VYNE-CyPA1-F2	5’ –	ATGTCTAACCCTAAGGTCTTCTTCG	–3’
VYNE-CyPA1-R2	5’ –	AGCCTGCTTTTTTGTACAAAGTTGG	–3’
VYCE-RIN4-F1	5’ –	AGAACGTTGTGCCATACCTCCAAGATCCTCCTCAGAAATC	–3’
VYCE-RIN4-F2	5’ –	AGAACGTTGTGCCATACCTCCAAGATCCTCCTCAGAAATC	– 3’
VYCE-RIN4-R1	5’ –	ATGGCACAACGTTCTCATGTACC	–3’
VYCE-RIN4-R2	5’ –	AGCCTGCTTTTTTGTACAAAGTTGG	–3’
Primers for Type III secretion system (T3SS) mutant<br>＊ The underlined 15-bp sequenced denote duplication for In-fusion			
T3SS-1F	5’–	CGGTACCCGGGGATCTGGAGCGAGTACGGCAATGT	–3’
T3SS-1R	5’–	TTTCTGTCCTGGCTGGATTGAGCCACGCTCACTCAT	–3’
T3SS-2F	5’–	AGTACCGCCACCTAATAAGAGGCAGCGCGATCGAA	–3’
T3SS-2R	5’–	CGACTCTAGAGGATCGCACTAGCCCTCTTGTGTTA	–3’
GmR-F	5’–	CAGCCAGGACAGAAATGCCT	–3’
GmR-R	5’–	TTAGGTGGCGGTACTTGGGT	–3’
Primers for qRT-PCR			
UBQ-F	5’–	ATGCAGATCTTCGTCAAGACCTTG	–3’
UBQ-R	5’–	ACCTCCCCTCAGACGAAG	–3’
RPS2-F	5’–	GGGAGTTTCAAATGGTGGAAGA	–3’
RPS2-R	5’–	CGGTTGTGCTGAAATTGGC	–3’
Chitinase-F	5’–	TCAATTTGCTCGGTCAATGGG	–3’
Chitinase-R	5’–	TCCCCATGCATTTGAAGCTT	–3’

Defensin-like-F	5'-	CATGGTCAGGGCCTTGTTTT	-3'
Defensin-like-R	5'-	CAAGCAAAACCAAAGCCCTG	-3'
ERN1-F	5'-	TGGACATGCCTAAGACTGATGGC	-3'
ERN1-R	5'-	TGAGCACAAGGGTGGAAGATCCCAC	-3'
NFR1-F	5'-	CCCTTGTACCACAGAACC	-3'
NFR1-R	5'-	GCTTTCTCTTCTTCCTTCTTCTG	-3'
RPG-F	5'-	AAGGAGAACTTATTAGCAAGAGAA	-3'
RPG-R	5'-	GTTGAATCTTGTCTCTCATTCATT	-3'
